# Identification of Circular RNAs and Their Targets in Leaves of *Triticum aestivum* L. under Dehydration Stress

**DOI:** 10.3389/fpls.2016.02024

**Published:** 2017-01-05

**Authors:** Yuexia Wang, Ming Yang, Shimei Wei, Fujun Qin, Huijie Zhao, Biao Suo

**Affiliations:** ^1^College of Life Sciences, Henan Agricultural UniversityZhengzhou, China; ^2^Department of Pathology, University of VirginiaCharlottesville, VA, USA; ^3^College of Food Science and Technology, Henan Agricultural UniversityZhengzhou, China

**Keywords:** wheat (*Triticum aestivum* L.), circRNAs, dehydration stress, deep sequencing, bioinformatics analysis

## Abstract

Circular RNAs (circRNAs) are a type of newly identified non-coding RNAs through high-throughput deep sequencing, which play important roles in miRNA function and transcriptional controlling in human, animals, and plants. To date, there is no report in wheat seedlings regarding the circRNAs identification and roles in the dehydration stress response. In present study, the total RNA was extracted from leaves of wheat seedlings under dehydration-stressed and well-watered conditions, respectively. Then, the circRNAs enriched library based deep sequencing was performed and the circRNAs were identified using bioinformatics tools. Around 88 circRNAs candidates were isolated in wheat seedlings leaves while 62 were differentially expressed in dehydration-stressed seedlings compared to well-watered control. Among the dehydration responsive circRNAs, six were found to act as 26 corresponding miRNAs sponges in wheat. Sixteen circRNAs including the 6 miRNAs sponges and other 10 randomly selected ones were further validated to be circular by real-time PCR assay, and 14 displayed consistent regulation patterns with the transcriptome sequencing results. After Gene Ontology (GO) and Kyoto Encyclopedia of Genes and Genomes (KEGG) pathway analysis of the targeted mRNAs functions, the circRNAs were predicted to be involved in dehydration responsive process, such as photosynthesis, porphyrin, and chlorophyll metabolism, oxidative phosphorylation, amino acid biosynthesis, and metabolism, as well as plant hormone signal transduction, involving auxin, brassinosteroid, and salicylic acid. Herein, we revealed a possible connection between the regulations of circRNAs with the expressions of functional genes in wheat leaves associated with dehydration resistance.

## Introduction

Wheat (*Triticum aestivum)* is the most widely grown and important food staple grain. During the life span of wheat, drought is a major environmental stress factor that affects wheat growth and development. Series of protective mechanism triggered by drought stress allow plants to adapt to adverse conditions (Wang et al., 2003). The response of plant to drought stress is potentially associated with the expression of protein-coding genes, such as the ones related to the elimination of reactive oxygen species (ROS) (Apel and Hirt, 2004), photosynthesis recovery (Wang et al., 2011), and stomatal adjustment (Miura et al., 2013). Along with the development of high-throughput sequencing technology and high-efficiency big data analysis, many more non-protein-coding genes are identified and characterized with response to environmental stress in plants. Previous studies have shown that non-protein-coding genes play vital roles in the stress response of wheat plant, including fungi-responsive long non-coding RNAs (lncRNAs) (Zhang et al., 2016) and dehydration responsive microRNAs (miRNAs) (Ma et al., 2015). The non-coding RNA itself, or interacting with other effectors as complex, is involved into the biochemical response directly or indirectly, regulating the expression of responsive functional genes at the transcriptional and/or translational levels (Zhang, 2015; Khaldun et al., 2016).

Circular RNAs (circRNAs) is a type of non-coding RNAs that belongs to the class of endogenous RNAs lacking 5′ or 3′ ends like miRNAs. Since the first report of the genomic-based evidence of circRNAs (Salzman et al., 2012), abundance of circRNAs has been observed in humans (Salzman et al., 2012), Archaea (Danan et al., 2012), *Caenorhabditis elegans*, and mice (Memczak et al., 2013). These reports suggested that certain number of circRNAs function as transcriptional regulators. However, in plants, only a few circRNAs have been observed by RNA-seq technology. Recently, using rRNA depleted RNA-seq (RibominusSeq) and followed anchor aligning, Ye et al. (2015) identified 12 037 and 6012 circRNAs in rice (*Oryza sativa*) and Arabidopsis (*Arabidopsis thaliana*), respectively. Meanwhile, Lu et al. (2015) reported 2354 circRNAs in *Oryza sativa* that were identified through deep sequencing and computational analysis of ssRNA-seq data. According to the limited sequencing data, compared with the reports in animals, plant circRNAs were found to be enriched for miniature inverted-repeat transposable elements (MITEs), revealing relatively less association with the repetitive regions and longer flanking introns (Sablok et al., 2016). However, there is no report to date about the sequence character of circRNAs in complex polyploid plant such as *Triticum aestivum* (AABBDD).

The current evidence in animals suggests that circRNAs act as miRNA sponges to competitively terminate suppression of their mRNA targets (Hansen et al., 2013). Therefore, the interactions between circRNA, miRNA, and mRNA have been considered as the main manner in the transcriptional and post-transcriptional regulation. The large-scale portal CircNet (Liu et al., 2015) has already been developed to reveal the regulatory role of circRNAs in circRNA-miRNA-gene regulatory networks. Similar to the results in animal, the functional expression of plant circRNAs has also been tightly linked to the tissue specificity (Liu et al., 2015). Moreover, the other's previous study in rice has also proposed that the circRNAs play an important role in regulating the response to environmental stress such as phosphate imbalance (Ye et al., 2015). To our knowledge, there is still no evidence showing the role of circRNAs in the stress resistance of wheat.

To explore the quantity of circRNAs in wheat and their potential function in the regulation of dehydration response, we first genome-widely identified circRNAs in wheat seedling leaves using high-throughput sequencing technology combined with the real-time PCR validation. Moreover, the sponge action of differentially expressed circRNAs and related mRNAs were further predicted and discussed using bioinformatics tools in present study.

## Materials and methods

### Plant materials and stress treatments

Seeds of wheat cultivars (*Triticum aestivum* L. cv. Aikang 58) were surface-sterilized with 1% NaClO for 10 min, and rinsed 3 times in distilled water for 2 min each. After soaking in tap water for 12 h, the seeds were allowed to germinate for 4 days in a dark incubator at 25°C. Five germinating seeds were placed into each plastic pot containing half strength Hoagland's nutrient solution in a phytotron. The environment was controlled at 25°/22°C (day/night) under a 12 h photoperiod, a 300 μmol m^−2^ s^−1^ of photosynthetic photon flux density (PPFD), and a relative humidity of 75 to 85%. At the two-leaf stage, the cups of wheat seedlings were randomly divided into two groups. The artificially dehydration-stressed group was induced by applying a final 20% polyethylene glycol (PEG) 6000 solution to achieve an osmotic potential of −0.975 MPa. The equal volume of Hoagland's nutrient solution was supplemented to another group serving for control treatment. Leaf tissues were harvested from both sets of seedlings 12 h after treatment. For each treatment, the representative leaves were retrieved from three independent pots of seedlings and combined as one sample set. Both samples were snap frozen in liquid nitrogen and stored at −80°C until use.

### Total RNA extraction, circRNA library construction, and sequencing

For RNA-seq-based comparative transcriptome analysis, it is recommended that the experimental design should include at least three biological replicates per treatment group (Auer and Doerge, 2010). The main objective of this study was to primarily screen the circRNAs in wheat leaves that differentially expressed under dehydration stress. With the purpose of minimizing the deviation between different parallel samples under the same treatment, the sample was retrieved from independent three plots of wheat seedlings. Furthermore, at least 2-fold (log2) change was used as cutoff, comparing commonly used 1-fold cutoff (Hivrale et al., 2016). Subsequently, the real-time PCR assay was used to validate the circularity of sequence and its expressional difference based on the examinations of three biological replicates. The three biological replicates in real-time PCR assay would make up for the deficiency of without biological replicates during RNA-seq analysis.

The total RNA was extracted from the control and PEG-stressed wheat leaves using Trizol (Invitrogen Corp., Carlsbad, CA, USA) followed by acid phenol chloroform extraction. Total RNA from each sample was used to prepare the circRNA sequencing library. Five micrograms of total RNA were pretreated to enrich circRNA using CircRNA Enrichment Kit (Cloud-seq Inc, USA). RNA libraries were constructed by using pretreated RNAs with TruSeq Stranded Total RNA Library Prep Kit (Illumina, San Diego, CA, USA) according to the manufacturer's instructions. Libraries were controlled for quality and quantified using the BioAnalyzer 2100 system (Agilent Technologies, Inc., USA). The libraries were denatured as single-stranded DNA molecules, captured on Illumina flow cells, amplified *in situ* as clusters and finally sequenced for 150 cycles on Illumina HiSeq Sequencer (USA) according to the manufacturer's instructions.

### Identification of circRNAs

Paired-end sequencing was conducted using Illumina HiSeq Sequencer after quality filtering using an in-house perl scripts. In this step, clean reads were obtained by removing low quality reads containing adapter and poly-N from raw data. The clean reads were aligned to the reference genome/transcriptome (IWGSC1.0+popseq) with BWA-MEM software (Li, 2013). The circRNAs were detected and identified by CIRI algorithm (version1.2) with genomic annotations from Ensembl Plants release 29 (Gao et al., 2015). The BWA-MEM alignment was performed using the parameters recommended by CIRI (bwa mem -T 19 genome_bwa_index sample_R1.fastq sample_R2.fastq 1>output.sam 2>output.log). The CIRI parameters for circRNAs identification was the default command line (CIRI_v1.2.pl -I input.sam -O output_circRNAs.txt -F genome.fa -P -A Ensembl_wheat29.gtf). The CIRI algorithms identified the circRNA candidates through a two-step filtering. The first step detects the paired chiastic clipping (PCC) signals in the Sequence Alignment Map (SAM) of BWA-MEM. The GT-AC signals were used as the PCC because they are the major splicing signals in eukaryotic transcription. Then, CIRI scans the SAM alignment again to detect additional junction reads and meanwhile performs further filtering to eliminate false positive candidates to remove false positives resulting from incorrectly mapped reads of homologous genes or repetitive sequences (Gao et al., 2015). Raw junction reads for all samples were normalized to total reads number and log2 transformed. Differentially expressed circRNAs were identified using fold change cutoff of 2 between the two treatment samples.

### Prediction of miRNA targets of circRNAs, mRNA targets of miRNA, and annotation of functions

CircRNA-miRNA-mRNA interactions, including the miRNA targets of differentially expressed circRNAs, as well as the mRNA targets of obtained miRNA, were predicted by psRNATarget (Dai and Zhao, 2011). The predicted mRNAs were blasted and functionally categorized according to Gene Ontology (GO) annotation by BLAST2GO software with the default parameters (https://www.blast2go.com/) (Conesa and Götz, 2008). The functions of predicted mRNAs were further analyzed by KAAS web-based automatic server with the default parameters (Moriya et al., 2007) (http://www.genome.jp/tools/kaas/). All the available plant genes databases were selected as the reference sequence sets for sequence homology analysis.

### Validation of differentially expressed circRNAs

Quantitative real-Time PCR (qRT-PCR) was used to validate the expression of circRNAs identified by RNA-seq. Sixteen differentially expressed circRNAs were used in experimental validation including the 6 circRNAs that predicted as miRNAs sponges, while the other 10 circRNAs were randomly selected from the differentially expressed ones. Two micrograms of total RNA were treated by DNase I (2270A, Takara, Japan) before real-time PCR quantification. The primers were designed using the “Out-facing” strategy to guarantee the amplifications were from circle template (Shen et al., 2015). The primer sequences are listed in Table 1. and the PCR products were validated by Sanger sequencing. The expression of circRNAs was quantified using SYBR green master mix (Applied Biosystems, Foster City, CA, USA) on an ABI StepOnePlus system (USA). Relative expression ratio (ΔΔ*Ct*) of each circRNA is calculated using 2^−^^Δ*ΔCt*^ method and presented in a log_2_ value, where the *Ct* is the threshold cycle value of the amplified target or reference gene (Livak and Schmittgen, 2001). After comparing with the expression of ACTB gene, the expression of housekeeping gene GAPDH was used as a reference for data normalization, because it displayed a more constant expression between the two treatment groups according to our pre-experiments. The real-time PCR assays were performed in three biological replicates and the results were expressed as mean ± standard deviation. Statistical analysis was conducted using the SPSS Statistics 18.0 software (IBM Corporation, NY, USA). Significant difference between PEG-treated and well-watered groups was compared using student *t*-test at 0.05 probability levels.

**Table 1:** **Primers in the real-time PCR assay for validating the differential expressions of circRNAs after sequencing**.

**Target name**	**Primer name**	**Sequence (5′ → 3′)**	**Amplicon size (bp)**	**Tm (°C)**
4A:214775737|215210034	1-Forward 1-Reverse	TTGCCTGTCTTGGGATCTTC GGCAAGGATCCCCCTAAATA	212	60.2 59.8
5B:221655648|222074380	2-Forward 2-Reverse	GTCGATCCTGCTCATGTTGA CCATTGTCGCATCTTGTTTG	215	59.8 60.1
5D:124926662|125373509	3-Forward 3-Reverse	AATAGGCGGAGAACCATTCA CCCCGACTCCTACTTGGTTA	158	59.0 59.4
5D:18963119|19091255	4-Forward 4-Reverse	TGGAGGAGTGGTTCTTCCAC CGTCGAAGGTGACGTACTTG	186	60.1 59.5
6A:201878372|202172929	5-Forward 5-Reverse	ACCTCTAGCCCCACTGTTGA CCAGGAGGCTTGAGAAAGAG	172	59.7 60.1
6D:169739848|170130661	6-Forward 6-Reverse	TTTTCATACCAAGGCAAGCA CTCCCTCATCATCTCCTCCA	201	60.2 60.1
2D:62914891|62916501	7-Forward 7-Reverse	AACTCGGTTTGAGATAAGCTACC CAAATTGAGCAACGCCAAAT	156	58.1 61.0
3B:15806226|15817694	8-Forward 8-Reverse	GCAGGACATGGAAGCTCATC GGCCAGGATCTTCTTCTTCC	185	60.1 60.0
3B:68150153|68152981	9-Forward 9-Reverse	ATTTACCCGAGGACCCACTT GATCCATGGCAACACTTCCT	227	59.7 59.9
IWGSC_CSS_4DS_scaff_2307013:2345|3583	10-Forward 10-Reverse	CCCATTTAAAAACTTGCGTGA GTGCCTTAACAGCCCTCTTG	193	60.0 59.9
7A:11715620|11717078	11-Forward 11-Reverse	TCCAAACATATGGAAGGATGC ACGGCTTCAGTTCAAACACC	192	59.8 60.2
5A:128534161|128535165	12-Forward 12-Reverse	AGGACCTTTGACGCACTGTT TCTCTATTCGCAGTCCATGCT	202	59.8 60.0
7D:12625708|12627116	13-Forward 13-Reverse	TCCAAACATATGGAAGGATGC AAATTCGAGAGGGGTTACCA	199	59.8 58.0
IWGSC_CSS_2BL_scaff_7985665:1994|3344	14-Forward 14-Reverse	AACCTTGTGCCATGGATGTA TCTCTATTCGCAATCCATGCT	175	58.9 59.9
IWGSC_CSS_5AL_scaff_2801994:5461|6705	15-Forward 15-Reverse	TGACATCAGCATCGCAAAAT TCAGCCTCTAACCAGCTTCC	205	60.2 59.6
6B:195302800|195307032	16-Forward 16-Reverse	TTGCGTTCAAGGCTAAAAGG TTTTTCCGGTTGATGAGGAC	198	60.4 59.9
GAPDH	CK-Forward CK-Reverse	ACATTAAGGGTGGTGCCAAG TGGTCATCAAACCCTCAACA	198	59.9 59.9

## Results and discussion

### Identification of circRNAs in wheat

**Figure 1:**
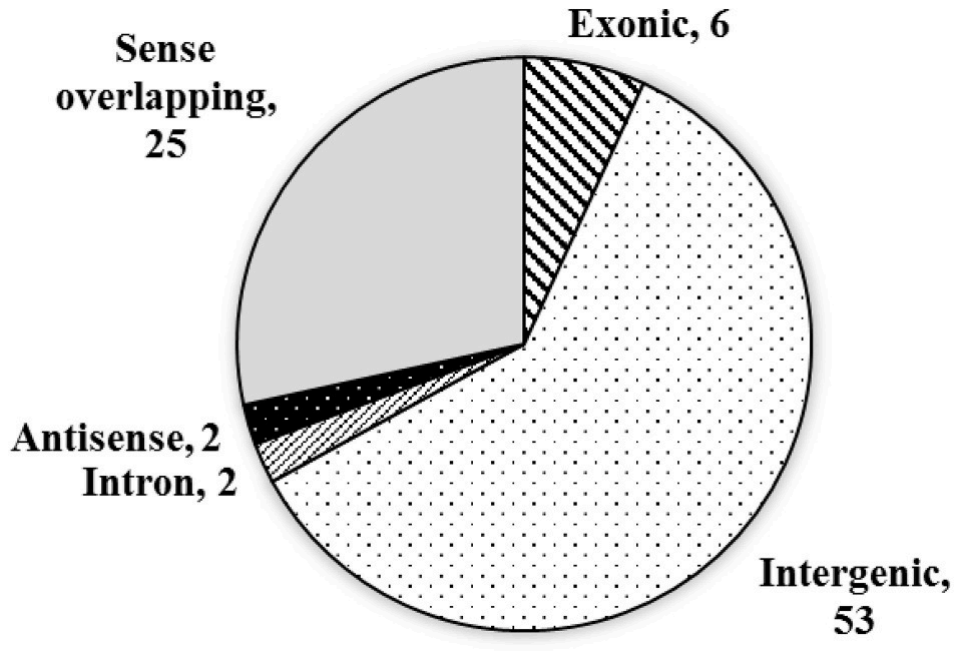
**The source and distribution of the circRNAs**.

Previously, although the sequences of circRNAs have been reported in some species (Sablok et al., 2016), circRNAs in wheat remains unknown. In present study, we extracted total RNA from PEG treated and well-watered wheat seedlings, respectively. After circRNAs enrichment, library construction, and sequencing, 52,484,890 and 38,345,455 raw reads, 7,872,733,500 and 5,751,818,250 nt total nucleotides were obtained from the two samples, respectively. The circRNAs were characterized by the 3′-5′ ligation in a splicing reaction of a single RNA molecule. Compared with traditional poly(A) based library sequencing, the circRNAs enriched library based sequencing improved the identification accuracy, although some circRNAs have been reported to be detected in libraries prepared from poly(A)-selected RNAs in human (Salzman et al., 2012). Based on the sequence reads, totally 88 circRNAs candidates were identified by the CIRI software in the two treatments of wheat leaves (Supplementary Table 1 and Data Sheet 1). Among them, six (6.8%) are exonic circRNAs, which were generated from exons of a single protein-coding gene, 53 (60.2%) of the circRNAs were generated from intergenic regions (intergenic circRNAs). Additionally, two (2.3%) of the circRNAs were generated by the intron, and another two (2.3%) were generated by the antisense strand of a single protein-coding gene, which were named intronic and antisense circRNAs, respectively. The remaining 25 (28.4%) of the circRNAs were generated from sense overlapping regions (Figure 1).

Comparing with previously limited reports on other higher plants, 12,037 (6074 exons) and 6012 (5152 exons) circRNAs were identified from *Oryza sativa* and *Arabidopsis thaliana*, respectively, by analyzing the publically available RNA-seq data (Ye et al., 2015), while 2354 circRNAs (1356 exons) were identified in rice through deep sequencing and computational analysis of ssRNA-seq data (Lu et al., 2015). A recently deep sequencing research in tomato identified 854 circRNAs (615 exons), in which 163 exhibiting chilling responsive expression (Zuo et al., 2016). The 88 identified circRNAs number and 6.8 percentages of exons were not as many as previous reports in other plants. The results should be attributed to following possible reasons: (i) The sequencing data is one of the most factor influencing the number of identified circRNAs, because more reads number denotes not only the high detection rate of circRNAs, but also could eliminate the false positives (Szabo and Salzman, 2016). The sequencing data size in present study is only 90 M reads, while it was 710 million paired-end reads sized 100 bp in the report on rice (Lu et al., 2015); (ii) The available genomic sequencing is limited in public database (http://plants.ensembl.org). According to the Ensembl Plant release 29 that used in the circRNAs screening, the known gene number of wheat is only 5610, while its genome size is 16000 Mb. Comparatively, there are 35679 available genes number with 120 Mb genome size for *Arabidopsis thaliana*, and 27655 available genes number with 389 Mb genome size for rice. The bigger genome size with only a few available genes number should be the main reason of low exon percentage; (iii) The software for the circRNAs prediction. We identified the circRNAs in wheat using CIRI algorithm (Gao et al., 2015). It is an efficient and unbiased algorithm for the *de novo* identification of circRNAs, which has already been used in some organizations including animals, humans, and plants (Hansen et al., 2016; Zuo et al., 2016). CIRI has been proven to be more useful when conducting more unbiased circRNA analyses or in poorly annotated organisms, comparing to other algorithms (Hansen et al., 2016). However, it is known that the differences between organization of genomes might influence the circRNAs identification results (Chen Y. E. et al., 2016), the specific algorithm for wheat circRNAs detection is still waiting for further comparison or development; (iv) The expression of circRNAs in higher plants often show tissue-, developmental-stage, or stress- specific expression patterns (Sablok et al., 2016). The wheat plant material used in present study was leaves when seedlings were exposed to dehydration stress. The implied possible mechanism deserves for further investigation from the aspect of circRNAs regulation.

### The differential expression patterns of circRNAs in wheat under dehydration stress

The specific expression of circRNAs implies possible associations with their biological functions. To investigate whether the circRNAs expressed in a specific manner in wheat under dehydration stress, we compared the circRNAs expression patterns between the wheat seedlings under PEG treatment and that under well-watered condition (control). The results showed that distinct difference patterns of circRNAs in both two wheat seedlings group were observed. Among the identified 88 circRNAs, 62 circRNAs showed significant difference between the PEG and control treatment groups, containing 16 upregulated circRNAs, and 46 downregulated circRNAs (Supplementary Table 2), which indicated their specific roles in the anti-dehydration stress regulation.

### Putative functions of the regulations of wheat circRNAs acting as miRNA sponges

The circRNAs have been reported to play important roles in regulating the functional genes expressions by acting as the miRNA sponges and preventing them from regulating their target mRNAs (Hansen et al., 2013). To evaluate whether wheat circRNAs could affect post-transcriptional regulation of functional genes by binding to miRNAs, the bioinformatics methods were employed to identify the circRNA-originating target mimics in wheat based on the differentially expressed circRNAs. We found that six of the 62 differentially expressed circRNAs had putative miRNA-binding sites (Table 2), and totally 26 miRNAs were predicted. Moreover, all the six differentially expressed circRNAs had three to eight miRNA-binding sites, which was similar to that reported in human (Memczak et al., 2013), but was significantly higher than that reported in rice (Lu et al., 2015) and tomato (Zuo et al., 2016).

**Table 2:** **The miRNAs corresponding to the differentially expressed circRNAs in wheat leaves under dehydration stress**.

**Number**	**Differentially expressed circRNAs**	**Corresponding miRNAs**
1	4A:214775737|215210034	tae-miR1127b-3p; tae-miR1120b-3p; tae-miR9679-5p
2	5B:221655648|222074380	tae-miR171a; tae-miR1133; tae-miR1134; tae-miR1137a
3	5D:124926662|125373509	tae-miR1118; tae-miR1129; tae-miR1130a; tae-miR1133; tae-miR1122b-3p; tae-miR1127b-3p; tae-miR1130b-3p
4	5D:18963119|19091255	tae-miR9667-5p; tae-miR9662b-3p; tae-miR1120b-3p; tae-miR1130b-3p; tae-miR6197-5p; tae-miR5049-3p; tae-miR9773
5	6A:201878372|202172929	tae-miR1138; tae-miR1122b-3p; tae-miR9667-5p; tae-miR5175-5p; tae-miR9778; tae-miR9781
6	6D:169739848|170130661	tae-miR1121; tae-miR1125; tae-miR1128; tae-miR1133; tae-miR9655-3p; tae-miR1130b-3p; tae-miR5049-3p; tae-miR9773

**Figure 2:**
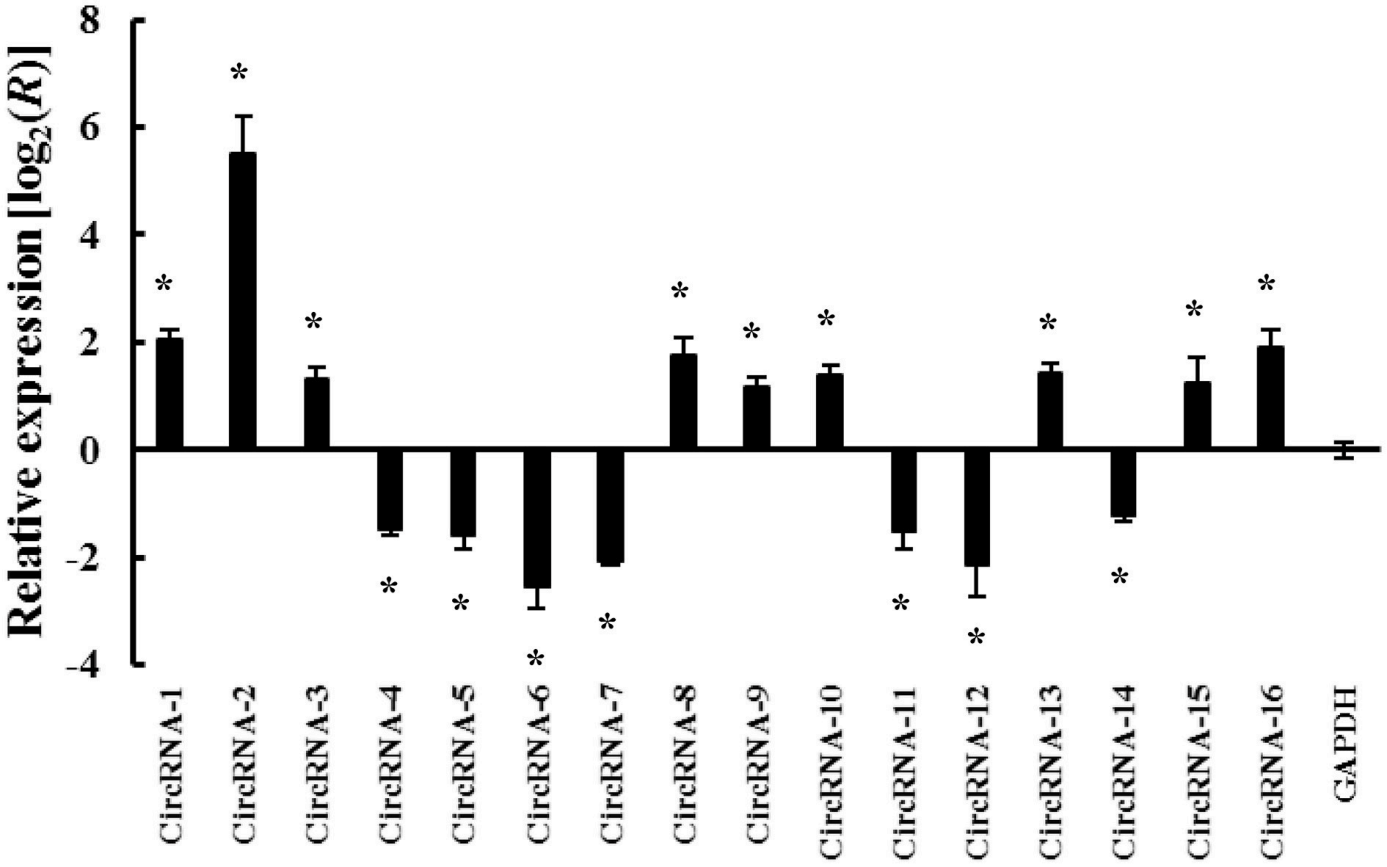
**Validation of the differentially expressed circRNAs by real-time PCR assay**. The primers were designed using the “Out-facing” strategy to guarantee the amplifications were from circle templete. Relative expression ratio (ΔΔ*Ct*) of each circRNA is presented in a log_2_ value. The expression of GAPDH gene was used the internal reference. circRNA-1, 4A:214775737|215210034; circRNA-2, 6D:169739848|170130661; circRNA-3, 5D:124926662|125373509; circRNA-4, 5D:18963119|19091255; circRNA-5, 5B:221655648|222074380; circRNA-6, 6A:201878372|202172929; circRNA-7, 2D:62914891|62916501; circRNA-8, 3B:15806226|15817694; circRNA-9, 3B:68150153|68152981; circRNA-10, IWGSC_CSS_4DS_scaff_2307013:2345|3583; circRNA-11, 7A:11715620|11717078; circRNA-12, 5A:128534161|128535165; circRNA-13, 7D:12625708|12627116; circRNA-14, IWGSC_CSS_2BL_scaff_7985665:1994|3344; circRNA-15, IWGSC_CSS_5AL_scaff_2801994:5461|6705; circRNA-16, 6B:195302800|195307032. The circRNAs from 1 to 6 were predicted as miRNAs sponges, while the other 10 circRNAs were randomly selected. The values are mean ± SE (*n* = 3). Means of PEG-treated samples denoted by the sign “^*^” significantly differ at *P* < 0.05 according to student *t*-test, comparing to that of well-watered control.

The circular structures and expression patterns of the 6 circRNAs were verified using real-time PCR assay followed by Sanger sequencing. The six genes were selected because they were predicted to have putative miRNA-binding sites, denoting they have more possibility to be involved in the dehydration response. The primers were designed using the “Out-facing” strategy that the linear mRNA will be excluded from amplification (Shen et al., 2015). As shown in Figure 2, the six circRNAs that were predicted as miRNA sponge showed positive amplicons from expected corresponding circular templets, while five displayed consistencies in expressional difference with the transcriptome sequencing results. Additionally, among the totally 16 tested circRNAs candidates, all were validated for circularity and 14 showed consistent expression patterns with RNA-seq results (Figure 2 and Supplementary Table 2). The accuracy of circRNA identified by deep sequencing was comparable with the 85.7% that previously reported in rice (Lu et al., 2015).

### Functional categorization of predicted mRNA

It has been reported that the miRNAs participate various plant physiological process under environmental stresses by regulating the functionally genes expressions (Zhang, 2015; Khaldun et al., 2016). To detect the possible role of wheat circRNAs in the resistance against to dehydration stress, the mimic miRNA of differentially expressed wheat circRNAs were further used to predict their possible target mRNAs by bioinformatics analysis. As shown in Supplementary Table 3, 539 mimic mRNAs were predicted from the 22 putative circRNAs-binding miRNA, the remaining four putative circRNAs-binding miRNA could not mimic target mRNA using psRNATarget online server.

**Figure 3:**
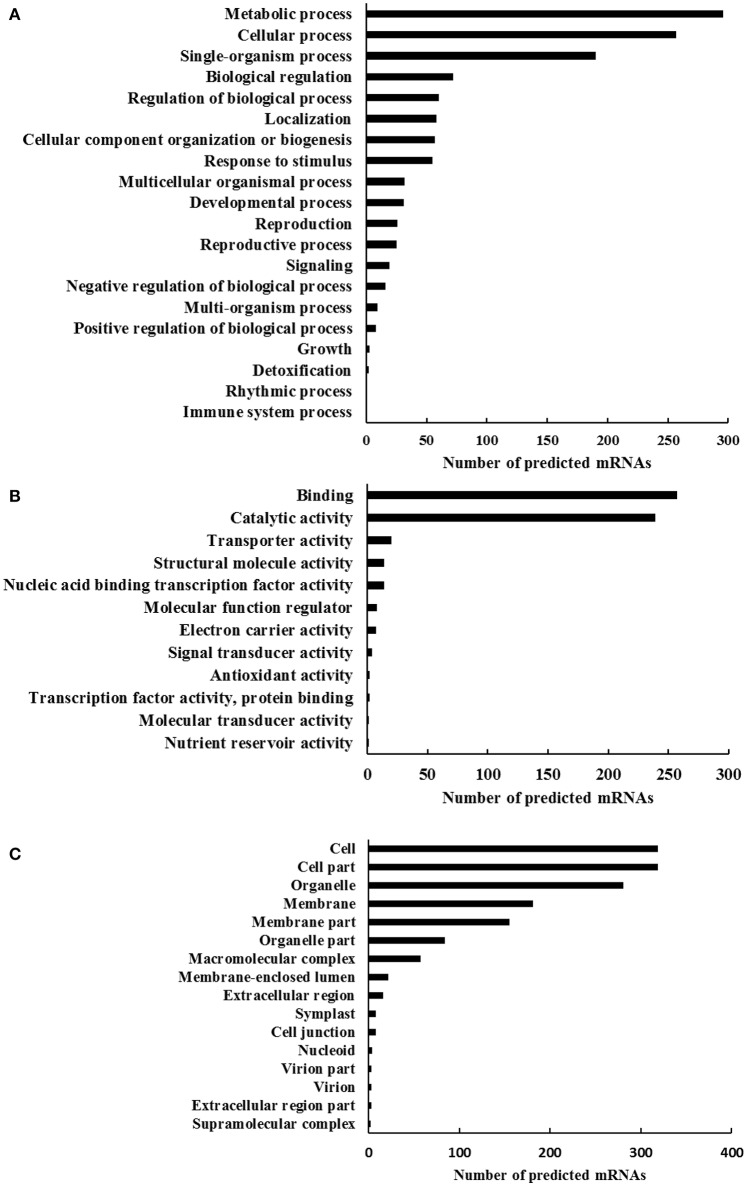
**The gene ontology profile of the mimic mRNA targets of the differentially expressed circRNAs in wheat seedlings leaves**. The bar charts represent the functional annotations of most relevant **(A)** biological processes, **(B)** molecular functions, and **(C)** cellular components until the second level of complexity.

To understand the genes function associated with the differentially expressed circRNAs, the GO analysis was performed by employing the BLAST2GO software. The GO functional categorization generated 843 annotations from the 539 predicted mRNAs (Figure 3). In that, totally 364, 407, and 386 mRNAs could be classified as the first level classifications of biological processes, molecular functions, and cellular components, respectively. Among the biological process classification, 296 and 257 mRNAs were classified into the categories of metabolic process (GO: 0008152) and cellular process (GO: 0009987), respectively (Figure 3A). Interestingly, 72 and 55 mRNAs were categorized as the categories of biological regulation (GO: 0065007) and response to stimulus (GO: 0050896), respectively. In the molecular functions classification, two main proportions were binding (GO: 0005488) and catalytic activity (GO: 0003824), which had 257 and 239 predicted mRNAs, respectively (Figure 3B). When the predicted mRNAs were classified according to the cellular component classification, categories cell (GO: 0005623) and cell part (GO: 0044464) both made up the largest proportion of 319 predicted mRNAs, followed by organelle (GO: 0043226) that had 281 predicted mRNAs (Figure 3C). The GO analysis on predicted mRNAs showed that the targets of differentially expressed circRNAs under dehydration stress were associated with various functions involving in different cellular components, biological process, and molecular functions. The differential expressions of circRNAs have already been reported in rice responding to Pi-starvation stress (Ye et al., 2015), and in tomato fruits suffering from chilling stress (Zuo et al., 2016), suggesting that circRNAs might play a role in responding to environmental stress. According to the report in rice (Lu et al., 2015), the mechanism of circRNAs participating in the stress response should be attributed that it exhibit alternative splicing circularization patterns and act as a negative regulator of their parental genes.

**Figure 4:**
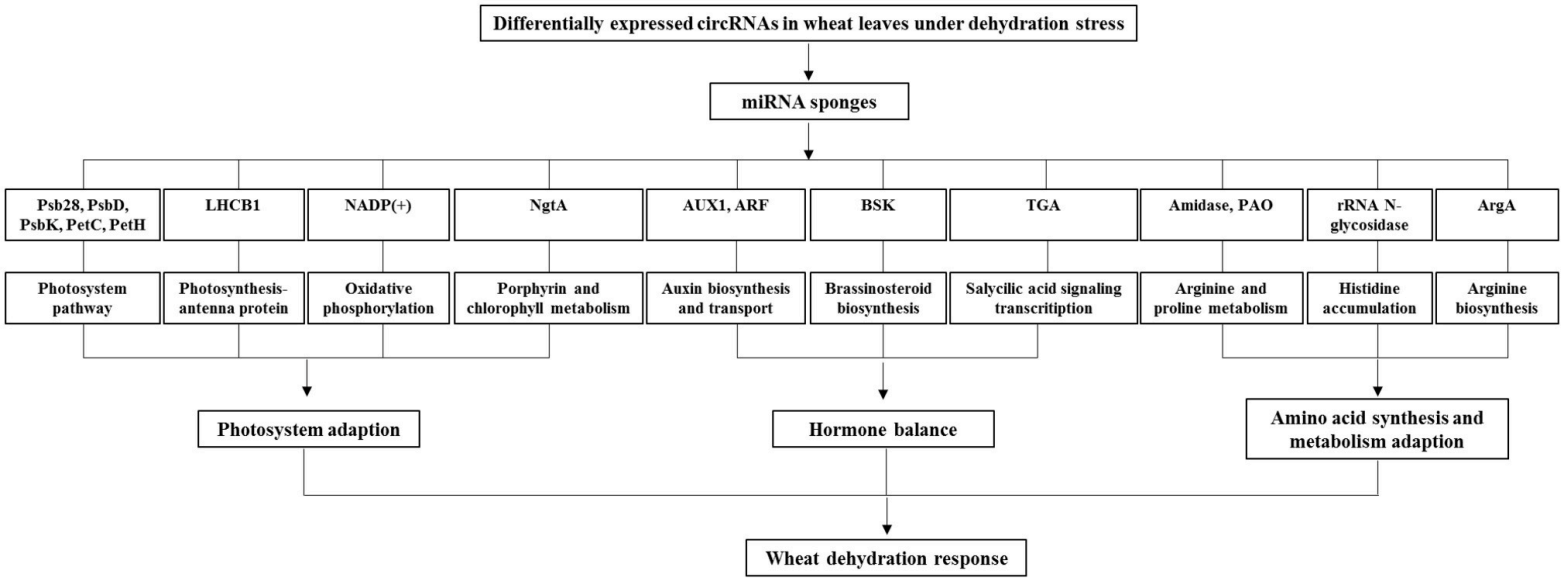
**Possible regulatory mechanism involving differentially expressed circRNAs and their target genes in wheat leaves under dehydration stress**. Psb28, photosystem II 13 kDa protein; PsbD, the photosystem I subunit II; PsbK, the photosystem I subunit X; PetC, cytochrome b6f complex iron-sulfur subunit; PetH, ferredoxin-NADP^+^ reductase; the light-harvesting complex II chlorophyll a/b binding protein 1 (LHCB1); Aux1, auxin influx carrier; ARF, auxin response factor; BSK, brassinosteroid signaling kinase; TGA, salicylic acid signaling transcription factor; NADP(+), NADPH dehydrogenase; PAO, polyamine oxidase; NgtA, 1-naphthol glucuronyltransferase; ArgA, amino-acid N-acetyltransferase.

The function of the predicted target mRNAs of circRNAs were further analyzed by KEGG pathway annotation method and 183 pathways were obtained. Among the obtained KEGG pathways, some of them are associated with the drought stress resistance in wheat or other higher plants. For example, as shown in Figure 4, the photosynthesis pathway related proteins included the photosystem II 13 kDa protein (Psb28), the photosystem I subunit II (PsbD), the photosystem I subunit X (PsbK), cytochrome b_*6*_f complex iron-sulfur subunit (PetC), and ferredoxin-NADP^+^ reductase (PetH). The photosynthesis–antenna proteins included the light-harvesting complex II chlorophyll a/b binding protein 1 (LHCB1). Interestingly, four predicted target mRNAs were involved in the plant hormone signal transduction pathway, including the auxin biosynthesis and transport associated auxin influx carrier (Aux1) and auxin response factor (ARF), the brassinosteroid biosynthesis associated BR-signaling kinase (BSK), as well as the salicylic acid signaling transcription factor TGA. Besides, the predicted target mRNAs of differentially expressed circRNAs also included the oxidative phosphorylation related NADPH dehydrogenase [NADP(+)], the arginine and proline metabolism related amidase and polyamine oxidase (PAO), the histidine accumulation related rRNA N-glycosidase, the porphyrin and chlorophyll metabolism related 1-naphthol glucuronyltransferase (NgtA), and arginine biosynthesis related amino-acid N-acetyltransferase (ArgA), etc.

The drought stress imposes serious physiological injury on higher plants including wheat. Plants have been evolved varieties of resistance capability against drought stress by regulating different physiological, photosynthetic, and molecular procedures. The circRNAs research in plant stress resistance has recently attracted interests because of their possible influence of functional genes (Sablok et al., 2016). In this study, the bioinformatics analysis results also suggested that the circRNAs in leaves involved in the dehydration resistance of wheat seedlings. The deduction could be supported by following two known regulation networks that have been proposed by others reports based on experimental data, including the photosynthesis- and hormone- regulation networks. Photosynthesis is considered as a very important indicator of drought stress, because it has been determined to be the most severe environmental factor that limits plant productivity in both natural and agricultural systems (Chen L. et al., 2016). Here we showed that the proteins in PSI, PSII, and photosynthesis—antenna play important roles during the resistance against drought stress, having consistency with previous reports (Chen L. et al., 2016; Rao and Chaitanya, 2016). Plant hormone participates in the drought stress resistance by mediating growth, development, nutrient allocation, gene expression, and source/sink transitions. Recently, the role of auxins in drought tolerance was postulated (Peleg and Blumwald, 2011), which was associated with the miRNA regulatory pathway (Ding et al., 2013). Brassinosteroid (BR) acts as a regulator to induce stomatal closure, which is similar to abscisic acid (ABA) (Acharya and Assmann, 2009). The exogenous application of BR was reported to protect the crop plants from stress injury by activating antioxidant enzymes, accumulating osmoprotectants, leading to the maintenance of photosynthesis activity, inducing the expression of stress-related genes, and other hormone responses (Peleg and Blumwald, 2011; Guo et al., 2013). Salicylic acid (SA) plays an important role as a signal molecule in drought stress tolerance. Its ability to induce a protective effect has been evidenced in both the genetic engineering plant of SA biosynthesis inhibition (Horváth et al., 2007), and the exogenously applied wheat plant under drought stress (Kang et al., 2013). Furthermore, genetic studies using various *Arabidopsis thaliana* mutants have demonstrated that SA exerts its role through coordinating interactions with other plant hormones including ABA (Santner et al., 2009), which is accepted to play a pivotal role in regulating plant drought resistance (Peleg and Blumwald, 2011). The present study proposed that the circRNAs might play a role in plant response to dehydration stress, by mediating the hormone signal pathway.

The rRNA N-glycosidase encoded by Traes_1DS_847CB932B.1, the putative target of 5D:124926662|125373509 through the sponge action of tae-miR1122c-3p, is histidine-tRNA cytoplasmic that involved in the metabolism of histidine. The accumulation of histidine has been determined to be important for plant response and adaptation to heavy metal stress (Sharma and Dietz, 2006). The rRNA N-glycosidase belongs to a kind of ribosome-inactivating proteins family that inhibit protein synthesis by depurinating rRNA, its regulation role has been suggested based on the expression profiles determination in *Oryza sativa* under various abiotic and biotic stresses including drought abuse (Jiang et al., 2008). Histidine is also involved in the signal transduction mediated by reactive oxygen species (Apel and Hirt, 2004). The present study inferred that the circRNAs regulated histidine accumulation might also involve the dehydration resistance of wheat seedlings.

Additionally, the functions of some other predicted mRNAs associated with the differentially expressed circRNAs have not been experimentally proven to involve in the dehydration resistance of plants until now. Their possible roles also deserved for attentions from the aspects of circRNAs regulation.

We did circRNAs enriched library based deep sequencing in wheat seedlings. Eighty-eight circRNAs candidates were identified while 62 were differentially expressed in dehydration-stressed seedlings compared to well-watered control. Among the dehydration-responsively expressed circRNAs, six were predicted to act as the corresponding miRNAs sponges in wheat. After bioinformatics analysis of the functions of the targeted mRNAs of miRNAs, the circRNAs were predicted to be involved in dehydration responsive process, such as photosynthesis, porphyrin, and chlorophyll metabolism, oxidative phosphorylation, amino acid biosynthesis, and metabolism, as well as plant hormone signal transduction, involving auxin, brassinosteroid, and salicylic acid. Herein, this study revealed a possible connection between the regulations of circRNAs with the expressions of functional genes in wheat leaves associated with dehydration resistance.

## Data deposition

All circRNA-seq data used in this research have been submitted to the National Center for Biotechnology information (NCBI) Sequence Read Archive (http://www.ncbi.nlm.nih.gov/Traces/sra) under the accession number GSE86641.

## Author contributions

YW carried out the experiments and wrote the manuscript. MY and BS analyzed the data. MY and SW assisted with doing the experiments. YW and BS conceived and designed the experiments. FQ and HZ helped to draft the manuscript and revise the manuscript. All authors read and approved the final manuscript.

### Conflict of interest statement

The authors declare that the research was conducted in the absence of any commercial or financial relationships that could be construed as a potential conflict of interest.
